# Supplementary material for: Dual Effects of Korean Red Ginseng on Astrocytes and Neural Stem Cells in Traumatic Brain Injury: The HO-1–Tom20 Axis as a Putative Target for Mitochondrial Function
Source: Cells. 2022 Mar 4;11(5):892. doi: 10.3390/cells11050892 (PMC8909112; doi:10.3390/cells11050892)
Supplement: Supplementary file 1 [file cells-11-00892-s001.zip › cells-1595099-supplementary.pdf]

Supplemenatry Figure S1

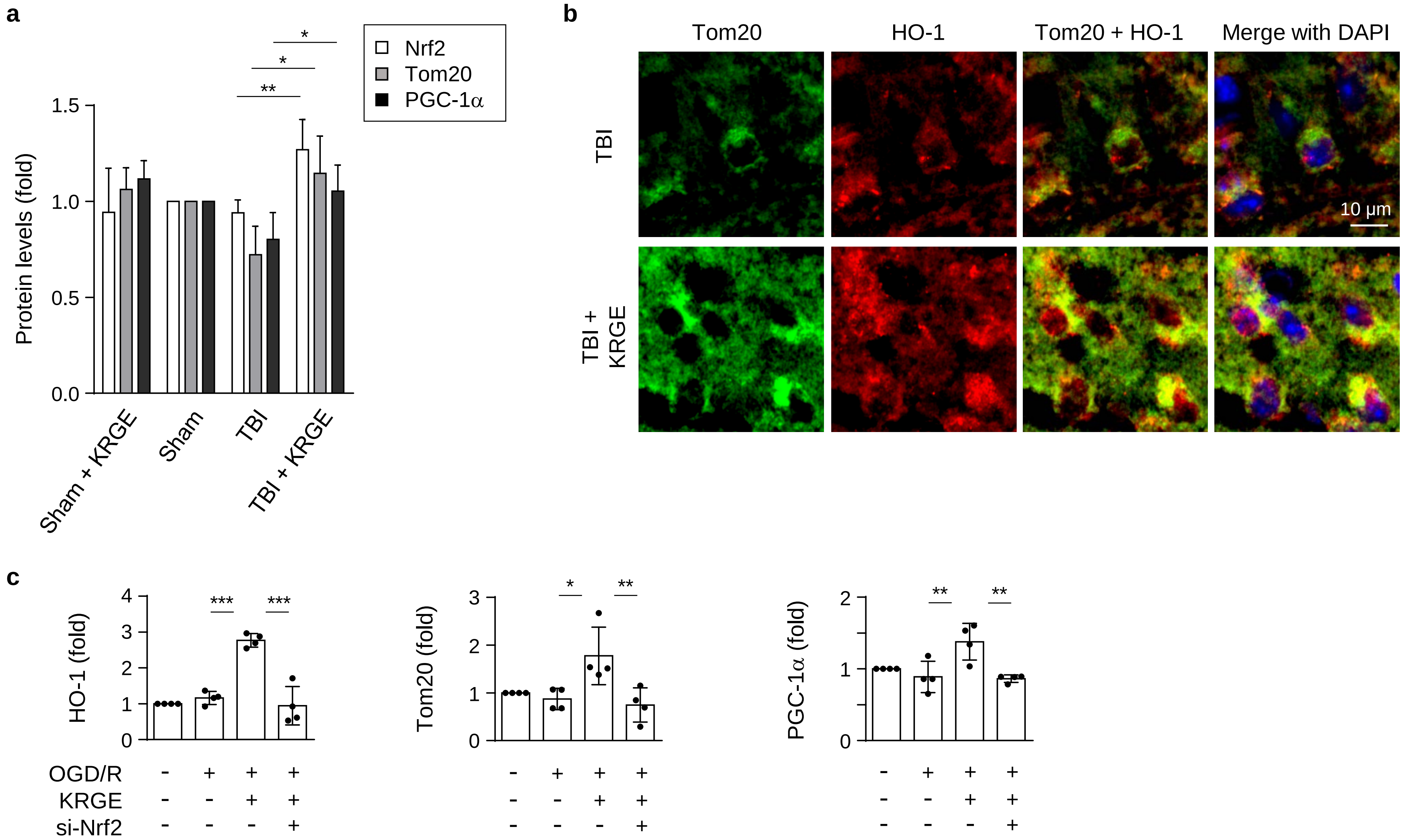

Supplementary Figure S1. Protein expression of HO-1 and Tom20. (a) Quantified graph obtained from brain tissues (approximately bregma -1 to -2), based on western blotting (*n* = 4 per each group). (b) Representative images of translocase of the outer membrane of mitochondria 20 (Tom20, green) and heme oxygenase-1 (HO-1, red) in a mouse brain obtained from traumatic brain injury (TBI) and TBI followed by Korean red ginseng extract (KRGE) (TBI + KRGE) treatment (*n* = 3 per group). 4',6-Diamidino-2-phenylindole (DAPI, blue) is used for nucleus detection (scale bar = 10 μm). (c) Quantified graph for Figure 1f is based on astrocyte cell lysate findings (n = 4 independent experiments). *P<0.05, **P<0.01, and ***P<0.001.

Supplementary Figure S2

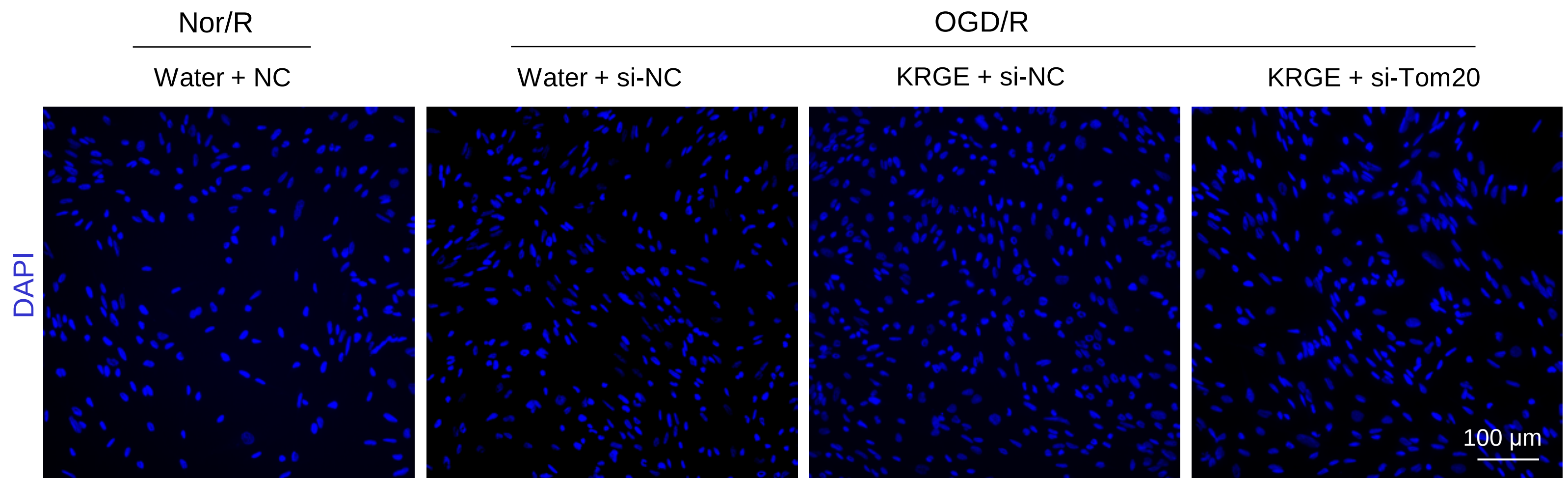

Supplementary Figure S2. DAPI images. Astrocytes on 12-well plates are transfected with siRNA for negative control (si-NC) or translocase of the outer membrane of mitochondria (si-Tom20), followed by oxygen-glucose deprivation/recovery (OGD/R) with or without Korean red ginseng extract (KRGE) treatment. Normoxia followed by recovery (Nor/R) is the control for OGD/R. Representative image of 4′,6-diamidino-2-phenylindole (DAPI, blue) staining in human astrocytes (*n* = 3 per group; scale bar = 100 μm).

Supplemenatry Figure S3

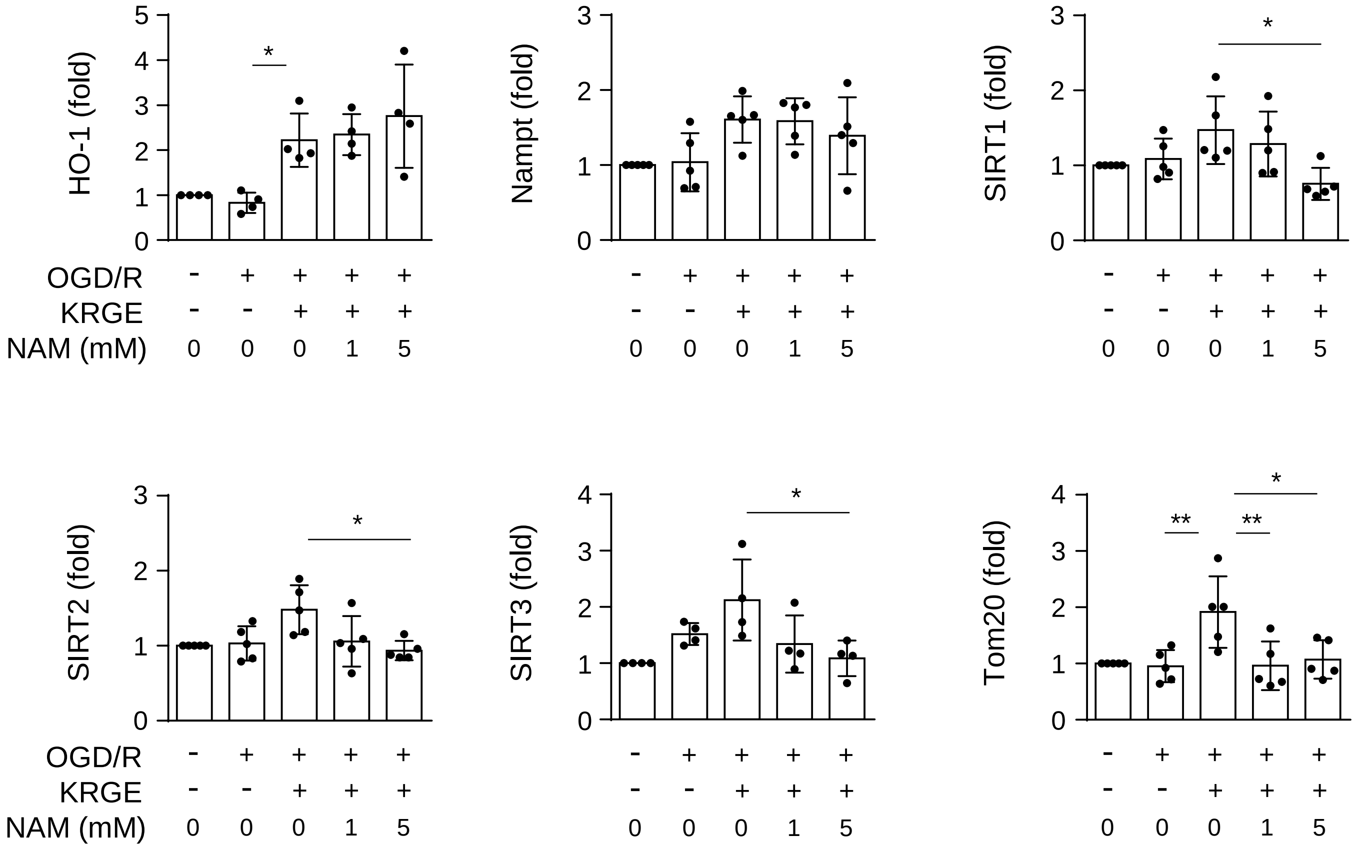

Supplementary Figure S3. Astrocytic Tom20 expression regulated by SIRT inhibitor. Astrocytes were subjected to 8 h oxygen-glucose deprivation (OGD), followed by recovery with 1 mM or 5 mM nicotinamide (NAM) plus Korean red ginseng extract (KRGE) for 24 h. Protein expressions in Figure 4e are quantified using ImageJ (GraphPad, San Diego, CA, USA) [e.g., heme oxygenase-1 (HO-1, $n = 4$); Nampt ($n = 5$); silent information regulator (SIRT1, $n = 5$); SIRT2 ($n = 5$); SIRT3 ($n = 4$); and translocase of the outer membrane of mitochondria 20 (Tom20, $n = 5$)]. *$P<0.05$ and **$P<0.01$.

Supplementary Figure S4

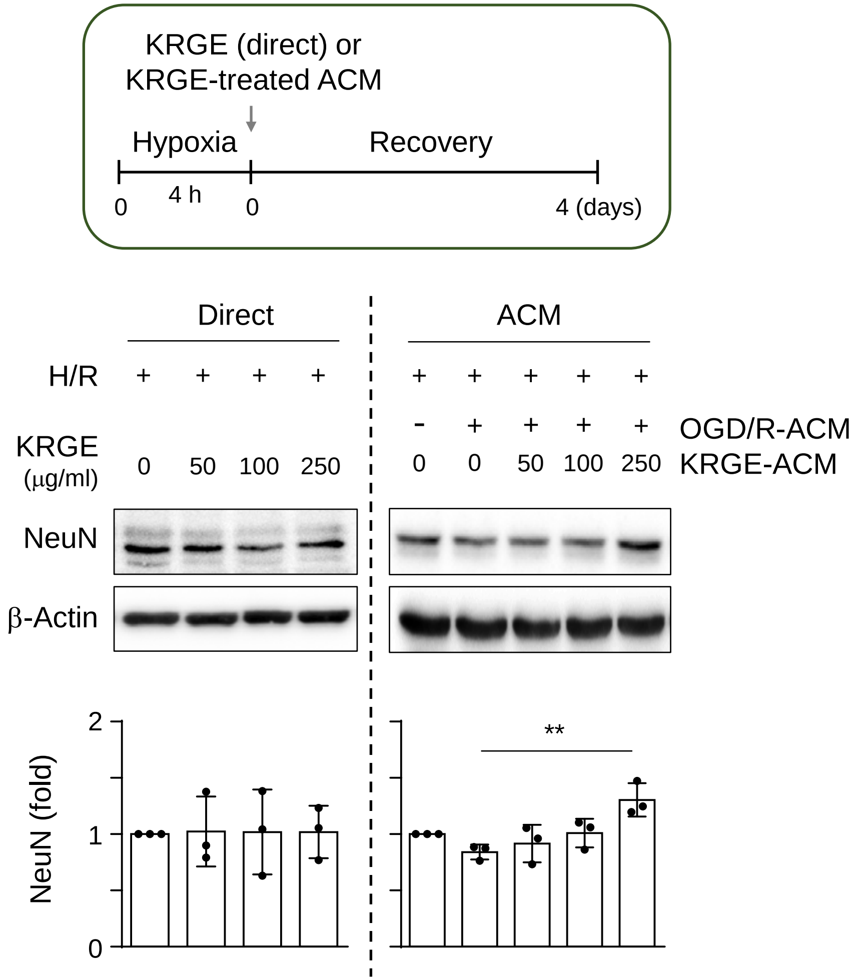

Supplementary Figure S4. Neuronal differentiation by KRGE. Neural stem cells (NSCs) are incubated in hypoxia for 4 h, followed by 4 days of recovery (H/R). In the recovery phase, the media are replaced with astrocytes conditioned media (ACM) and differentiation media in a 1:1 ratio. Indicated protein levels are assessed by western blotting (n =3 independent experiments). **P<0.01.
